# The role of autophagy in the midgut epithelium of Parachela (Tardigrada)

**DOI:** 10.1007/s00435-018-0407-x

**Published:** 2018-05-10

**Authors:** M. M. Rost-Roszkowska, K. Janelt, I. Poprawa

**Affiliations:** Department of Animal Histology and Embryology, University of Silesia in Katowice, Bankowa 9, 40-007 Katowice, Poland

**Keywords:** Digestive system, Ultrastructure, Cell death, Midgut

## Abstract

The process of cell death has been detected in the midgut epithelium of four tardigrade species which belong to Parachela: *Macrobiotus diversus, Macrobiotus polonicus, Hypsibius dujardini* and *Xerobiotus pseudohufelandi*. They originated from different environments so they have been affected by different stressors: *M. polonicus* was extracted from a moss sample collected from a railway embankment; *M. diversus* was extracted from a moss sample collected from a petrol station; *X. pseudohufelandi* originated from sandy and dry soil samples collected from a pine forest; *H. dujardini* was obtained commercially but it lives in a freshwater or even in wet terrestrial environment. Autophagy is caused in the digestive cells of the midgut epithelium by different factors. However, a distinct crosstalk between autophagy and necrosis in tardigrades’ digestive system has been described at the ultrastructural level. Apoptosis has not been detected in the midgut epithelium of analyzed species. We also determined that necrosis is the major process that is responsible for the degeneration of the midgut epithelium of tardigrades, and “apoptosis–necrosis continuum” which is the relationship between these two processes, is disrupted.

## Introduction

Tardigrades, which are commonly known as water bears, are small invertebrates that are closely related to Arthropoda and Onychophora. They are able to survive in extreme environments that can be lethal for other organisms (e.g., a lack of water, temperatures close to absolute zero, temperatures above 150 °C, X-ray irradiation) (Jönsson et al. 2005; Wełnicz et al. 2011; Rebecchi 2013; Czerneková et al. 2017; Jönsson and Wojcik 2017) due to their ability to undergo cryptobiosis (Møbjerg et al. 2011; Nelson et al. 2015). However, they are also suspected of having different mechanisms that play an important role in maintaining homeostasis and surviving (Schill et al. 2009). One of the organs in an animal’s body that forms a barrier against any stressors that could enter the organism along with nutrients is the middle region of the digestive system—the midgut (Teixeira et al. 2013). As it is known, stressors can enter the animal’s body through the epidermis or the digestive system with nourishments. In the digestive system, stressors can affect the digestive cells and cause irreversible alterations. However, different mechanisms (e.g., apoptosis, autophagy and/or necrosis) can counteract and participate in maintaining homeostasis. The most important feature is the relationship between these processes that can act as survival factors or can cause death (Malagoli et al. 2010; Franzetti et al. 2012). During our previous studies on the midgut epithelia of tardigrades, we studied the ultrastructure of the digestive and regenerative cells. As the material for these studies, we selected tardigrades that belong to Parachela (Eutardigrada): *Macrobiotus diversus, Macrobiotus polonicus, Hypsibius dujardini* and *Xerobiotus pseudohufelandi*. These species live in different environments: *M. polonicus* and *M. diversus* live in mosses (our specimens were collected from a polluted environment), *X. pseudohufelandi* originates from a dry terrestrial habitat, while *H. dujardini* lives in mosses in damp, shady areas as well as in freshwater habitats. The midguts of *M. polonicus, M. diversus, H. dujardini* and *X. pseudohufelandi* are lined with a simple epithelium that is composed of the digestive cells, which are the principal cells. At the anterior end of the midgut at the border with the foregut, a group of regenerative cells was observed in *M. polonicus, M. diversus* and *X. pseudohufelandi*. In *H. dujardini*, the regenerative cells formed an additional group at the border with the hindgut at the posterior end of the midgut. The precise structure and ultrastructure of the regenerative and digestive cells of *H. dujardini* and *X. pseudohufelandi* were presented in our previous papers (Rost-Roszkowska et al. 2013a; Hyra et al. 2016). The emphasis on the cell death of four species of Tardigrada (Parachela) has been discussed in this paper. We focused our attention on the different environments that the animals live in and the stressors that can affect the animals and that disrupt the maintenance of an organism’s homeostasis (e.g., starvation, lack of water and xenobiotics). Therefore, we have made the following hypotheses: (1) the autophagy is a selective or non-selective process; (2) the autophagy is responsible for cell protection; (3) apoptosis and/or necrosis are common processes of cell death in the midgut epithelium of tardigrades; (4) a crosstalk between autophagy and apoptosis and/or necrosis in the digestive system of tardigrades appears.

## Materials and methods

We selected four species of tardigrades belonging to order Parachela: *M. polonicus, M. diversus, X. pseudohufelandi* and *H. dujardini* as the material for our study. Specimens of *M. polonicus* were extracted from a moss sample that was collected from a railway embankment in Poznań. *M. diversus* was extracted from moss samples that were collected from a petrol station near Poznań and from the Poznań–Ławica airport. Specimens of *X. pseudohufelandi* were extracted from sandy soil samples collected from a pine forest on the Morasko University Campus, Poznań, using standard methods (Dastych 1980). Specimens of *H. dujardini* (Hypsibiidae) were obtained commercially from SCIENTO (UK).

### Light and electron microscopy

Twenty-five adult specimens of each analyzed species were fixed with 2.5% glutaraldehyde buffered with 0.1 M phosphate buffer (pH 7.4) (24 h at 4 °C) and postfixed with 2% OsO4 in a 0.1 M phosphate buffer (2 h at room temperature). Dehydration and embedding were performed as described earlier (Rost-Roszkowska et al. 2013a, b; Poprawa et al. 2015). Semi- and ultrathin sections were cut on a Leica ultracut UCT25 ultramicrotome. Ultrathin sections (50 nm thick), which were mounted on the formvar-covered grids (50 mesh), were stained with uranyl acetate and lead citrate (Reynolds 1963) and examined using a transmission electron microscope (Hitachi H500 at 75 kV).

### TUNEL assay (detection of cell death)

Ten adult specimens of each analyzed species were punctured with a thin Wolfram needle, incubated in a permeabilization solution (0.1% sodium citrate) (2 min on ice in 4 °C), washed in TBS (3 × 5 min) and stained with a terminal deoxynucleotidyl transferase dUTP nick end labeling (TUNEL) reaction mixture (In Situ Cell Death Detection Kit, TMR red, Roche; 60 min at 37 °C in the dark). A negative control was prepared according to the labeling protocol. The material was analyzed using an Olympus FluoView FV 1000 confocal microscope. Excitation at 595 nm was provided by a multi-line argon laser.

## Results

**Fig. 1:**
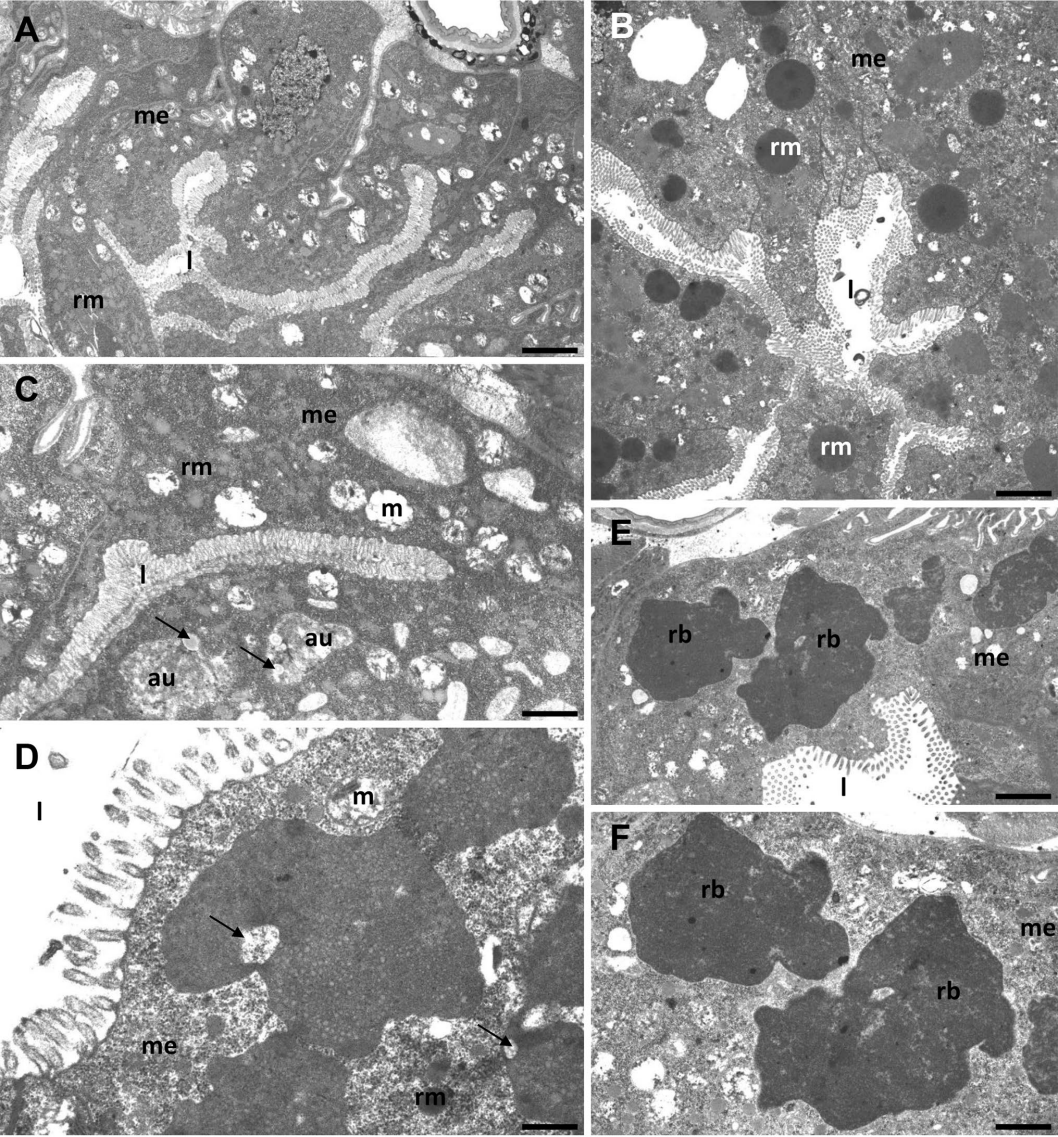
Midgut of *Macrobiotus polonicus* (**a, c, e, f**) and *Macrobiotus diversus* (**b, d**). **a** Cells of the midgut epithelium (me) poor in small spheres of reserve material (rm), midgut lumen (l), bar = 1.9 µm. **b** Cells of the midgut epithelium (me) with average amount of reserve material (rm), midgut lumen (l), bar = 2.6 µm. **c** Mitochondria with lost crista and electron-lucent content (m) in the cells of the midgut epithelium (me), autophagsome (au), midgut lumen (l), mitochondria (m), reserve material (rm), spheres of reserve material neutralized inside autophagosomes (arrow), bar = 1.2 µm. **d** Mitophagy (arrow) in the cells of the midgut epithelium (me), midgut lumen (l), mitochondria (m), reserve material (rm), bar = 0.6 µm. **e, f** Residual bodies (rb) in the cells of the midgut epithelium (me), midgut lumen (l), **e** bar = 1.4 µm, **f** bar = 1.2 µm

**Fig. 2:**
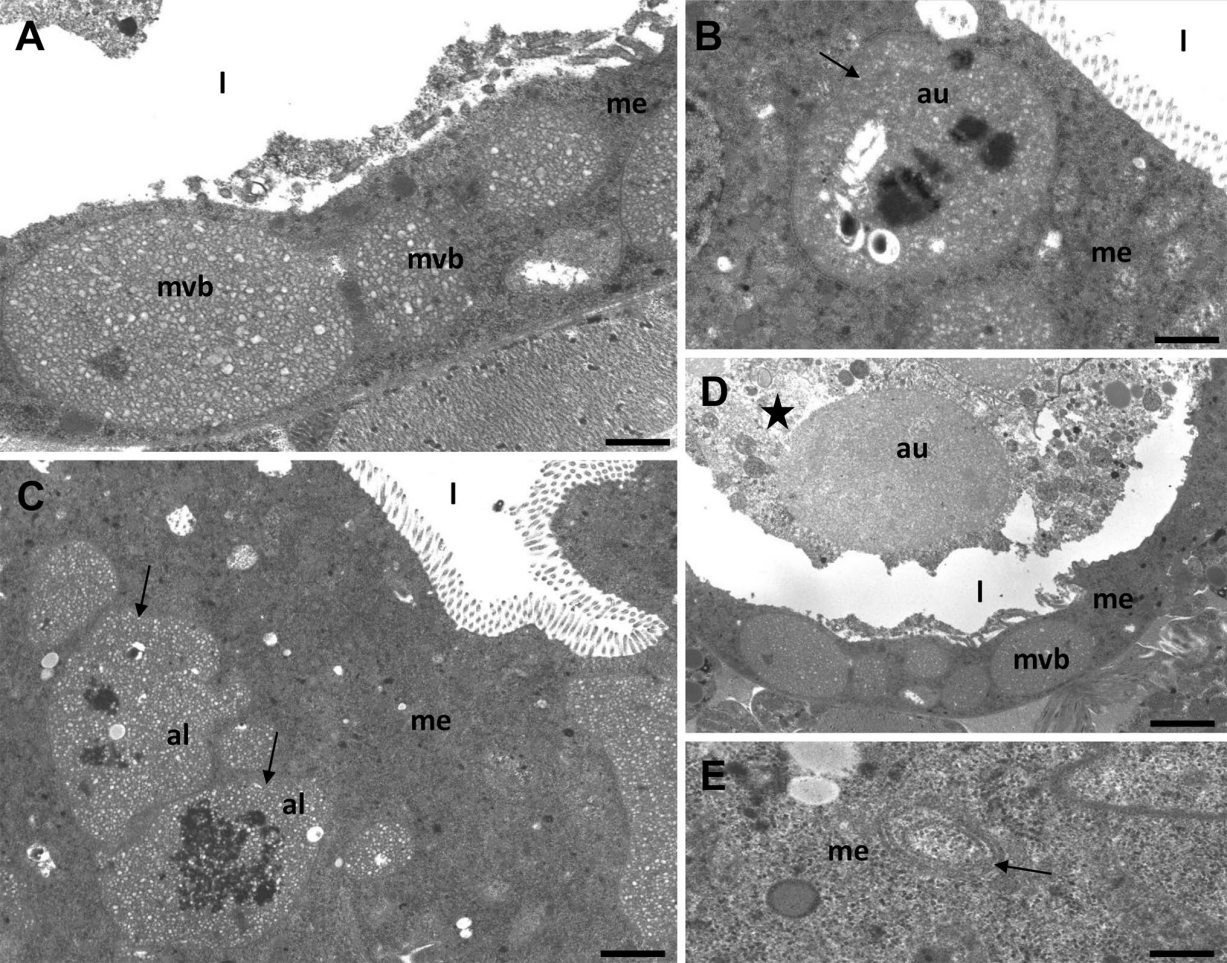
Midgut of *Xerobiotus pseudohufelandi.* **a** multivesicular bodies (mvb) in the cells of the midgut epithelium (me), midgut lumen (l), bar = 0.6 µm. **b** Multivesicular body (arrow) enclosed inside autophagosome (au), midgut epithelium (me), midgut lumen (l), bar = 1 µm. **c** Multivesicular body (arrow) enclosed inside autolysosome (al), midgut epithelium (me), midgut lumen (l), bar = 1.6 µm. **d** Digestive cells with large autophagosomes (au) died in a necrotic manner (asterisk), midgut epithelium (me), multivesicular body (mvb), midgut lumen (l), bar = 2.3 µm. **e** Formation of a phagophore (arrow), midgut epithelium (me), bar = 0.4 µm

**Fig. 3:**
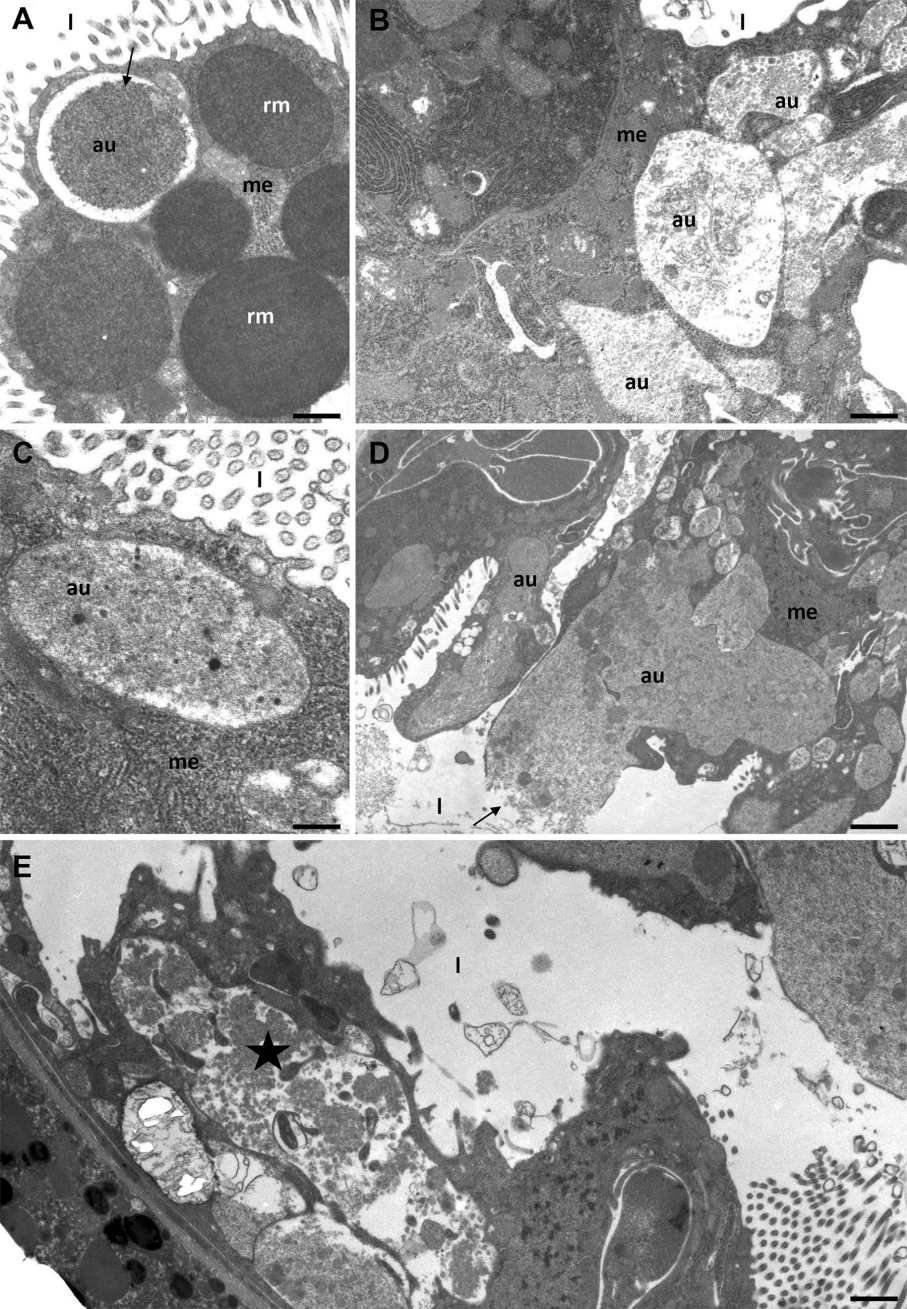
Midgut of *Hypsibius dujardini*. **a** Spheres of the reserve material (arrow) enclosed inside autophagosomes (au), midgut lumen (l), midgut epithelium (me), reserve material (rm), bar = 0.75 µm. **b, c** Autophagosomes (au) in the cells of the midgut epithelium (me) and midgut lumen (l). **b** Bar = 0.7 µm. **c** Bar = 0.3 µm. **d** Autophagosomes (au) in the cells of the midgut epithelium (me), midgut lumen (l), broken apical cell membrane (arrow), bar = 1.4 µm. **e** Digestive cells dying in a necrotic manner (asterisk), midgut lumen (l), bar = 1.1 µm

The process of autophagy was detected only in the cytoplasm of the digestive cells in the midgut epithelium of the analyzed species (Figs. 1a–f, 2a–e, 3a–e), while the regenerative cells showed no signs of autophagy. In all of the species studied, the formation of a double-membraned structure called a phagophore appeared as the first step of autophagy. After the closure of the blind ends of the phagophore (Fig. 2e), an autophagosome with organelles/structures enclosed inside was observed (Figs. 1c, 2b, 3a–d). The fusion of the autophagosome with a lysosome caused the formation of an autolysosome (Fig. 2c). As the final step of autophagy, residual bodies with an electron-dense content of the digested organelles were observed (Fig. 1e, f). When too many autophagosomes, autolysosomes and/or residual bodies appeared in the digestive cells, their cytoplasm began to be electron lucent and the number of organelles decreased gradually (Figs. 2d, 3e). The process of necrosis was activated. Eventually, the apical cell membrane broke and the cytoplasm along with the remains of the organelles was discharged into the midgut lumen (Fig. 3d) where they were digested. Apoptosis was not observed in the midgut epithelium of any of the species examined here. A TUNEL assay confirmed this observation. Therefore, in all of the species examined here, we detected the crosstalk between the autophagy and necrosis. Depending on the environment the animal lives, we observed different types of autophagy, because it was activated in the digestive cells depending on different stressors. The tardigrade species that were collected from the polluted environment (*M. polonicus* was collected from a railway embankment, *M. diversus* was collected from a petrol station) had digestive cells that were poor in small spheres of reserve material (*M. polonicus*) (Fig. 1a) or that had an average amount of reserve material (*M. diversus*) (Fig. 1b). In both these species, numerous degenerated organelles (mainly the mitochondria) accumulated in the entire cytoplasm (Fig. 1c, d). Mitochondria lost cristae and had an electron-lucent content. Their accumulation caused the appearance of double-membraned structure called as the phagophore which gradually surrounded mitochondria. In this case, numerous digested or transferred organelles and structures could be observed inside the autophagosomes. However, the mitophagy was the main type of autophagy observed in both tardigradan species (Fig. 1d). Additionally, some spheres with the reserve material were also neutralized inside the autophagosomes (Fig. 1c). In some specimens, large residual bodies were observed as the final step of autophagy (Fig. 1e, f). When the cytoplasm was rich in autophagic structures (autophagosomes, autolysosomes and/or residual bodies), the digestive cells died in a necrotic manner.

In the tardigrades that originated from the dry terrestrial environment (*X. pseudohufelandi*), large multivesicular bodies were detected (Fig. 2a) during our previous studies. However, in periods of lack of water, these multivesicular bodies were enclosed inside the autophagosomes (Fig. 2b) and the process of their digestion began inside autolysosomes (Fig. 2b, c). Eventually, the digestive cells with numerous autophagosomes or autolysosomes died in a necrotic manner (Fig. 2d).

In *H. dujardini*, spheres of the reserve material accumulated in the cytoplasm of the digestive cells (Fig. 3a). During periods of starvation when the animal did not feed itself, numerous spheres were enclosed inside the autophagosomes (Fig. 3a). Eventually, the cytoplasm was filled with large autophagosomes or autolysosomes (Fig. 3b, c). The apical cell membrane broke (Fig. 3d) and the organelles were discharged into the midgut lumen.

## Discussion

Cell death can be activated by many stressors that originate from the external environment and this process participates in the elimination of damaged or unwanted cells from an animal’s body (Teixeira et al. 2013; Wilczek et al. 2014; Lipovšek and Novak 2015). Therefore, it is responsible for, e.g., controlling the number of cells in the organs and protecting the organs against inflammation. Numerous types of cell death are known (e.g., entosis, anoikis, paraptosis, etc.). However, three types are best known: apoptosis, necrosis and autophagy. They are common processes that occur in the digestive epithelia of invertebrates (Malagoli et al. 2010; Franzetti et al. 2012; Romanelli et al. 2014, 2016; Wilczek et al. 2014; Lipovšek and Novak 2015; Rost-Roszkowska et al. 2015, 2016; Sonakowska et al. 2016). When the cytoplasm is rich in autophagic structures, apoptosis and/or necrosis is activated (Levine and Yuan 2005; Giusti et al. 2007; Tettamanti et al. 2007; Rost-Roszkowska et al. 2013b). Two types of cell death were observed in all of the Tardigrada species that were examined here: autophagy and necrosis. Initially, when a stressor is weak, autophagy is activated. However, when a stressor is too strong, autophagy initiates necrosis. A similar phenomenon was observed in the midgut epithelium of the tardigrade *I. g. granulifer*, which had been infected with microsporidia (Rost-Roszkowska et al. 2013b). When the cytoplasm of digestive cells in this species is rich in autophagic structures (autophagosomes, autolysosomes, and residual bodies) with microsporidia, necrosis is activated. During necrosis, when the membranes of the necrotic cell break, the cytoplasm is discharged into the midgut lumen along with pathogens and remaining organelles and is finally digested (Rost-Roszkowska et al. 2013b). Therefore, we can state that autophagy initially plays the role of a survival factor, but that eventually it causes cell death. The midgut lumen is the place where stressors that originate from the external environment can enter an organism, and the cells that form its epithelium protect the entire organism. Therefore, only cells that reach the midgut lumen can be affected by a stressor (Wilczek et al. 2014; Włodarczyk et al. 2017). In all of the tardigrades examined here, autophagy and necrosis were only detected in the digestive cells of the midgut epithelium, while they were not observed in the cytoplasm of the regenerative cells as has been described for other invertebrates (Tettamanti et al. 2007; Rost-Roszkowska et al. 2015, 2016). Apoptosis was not observed in the midgut epithelium of any of the species examined here. However, we have observed apoptosis in the gonads of *Isohypsibius granulifer granulifer* (Poprawa et al. 2015), *H. dujardini, Thulinius ruffoi* and *Richtersius coronifer* (our studies, data not published). Therefore, we can state that apoptosis is a process that is common for tardigrade tissues. The lack of this process in the digestive epithelium may be connected with the fact that apoptosis requires much more energy than necrosis. Apoptosis is an energy-dependent process (Zeiss 2003; Elmore 2007) and, therefore, involving necrosis in cell degeneration in the midgut epithelium of tardigrades provides the cells with energy efficiency.

In all of the tardigrades analyzed here, we observed that cell death was activated by different stressors. *M. polonicus* and *M. diversus* were collected from polluted environments: *M. polonicus* was extracted from a moss sample that was collected from a railway embankment in Poznań, while *M. diversus* was extracted from moss samples that were collected from a petrol station near Poznań and from the Poznań–Ławica airport. In both of these species, the process of mitophagy was detected. Mitophagy is a type of selected autophagy in which mainly damaged or non-active mitochondria are neutralized and discharged from the cytoplasm (Włodarczyk et al. 2017). Selective autophagy, in contrast to a non-selective process, forces the specific structures or organelles into the autophagosomes to cause them to be eventually digested (Mijaljica et al. 2012; Shaid et al. 2013; Romanelli et al. 2014, 2016). Depending on the organelles that are enclosed inside autophagosomes, we can distinguish mitophagy, reticulophagy, lipophagy, nucleophagy, etc. (Narendra et al. 2009; Mijaljica et al. 2012). Mitochondria are responsible for, e.g., the production of the energy that is delivered to all of the organelles or the release of the apoptogenic factors, the activation of the cell death, and finally, the maintenance of homeostasis (Klionsky and Emr 2000; Faron et al. 2015). Therefore, mitophagy is treated as the mechanism that protects cells from death (Xue et al. 2001; Youle and Narendra 2001; Narendra et al. 2009; Shaid et al. 2013; Faron et al. 2015; Włodarczyk et al. 2017). Oxidative stress is caused by many xenobiotics that originate from the external environment. It causes mitochondrial dysfunction because mitochondria are both the generators and targets for reactive species (Murphy 2009; Lee et al. 2012). It is highly likely that mitochondria are targeted to autophagosomes due to the accumulation of reactive species in *M. polonicus* and *M. diversus* that had been exposed to a polluted environment, and thus a cell is protected against death. When the cytoplasm is filled with numerous autophagic structures, necrosis is involved in a cell’s degeneration.

Because *X. pseudohufelandi* lives in a very dry environment, numerous multivesicular bodies have been described as the sources of water. Additionally, several small spheres with reserve material appeared. They mainly accumulated lipids, while polysaccharides and proteins were sporadic (Rost-Roszkowska et al. 2013a). The crosstalk between autophagosomes and multivesicular bodies is a common process in which the fusion of these structures causes the formation of amphisomes. After the fusion of amphisomes with lysosomes, their content is digested (Dunn 1994; Fader and Colombo 2009). In *X. pseudohufelandi*, when there is a lack of water, the multivesicular bodies are enclosed inside the autophagosomes and are probably utilized as the source of water. Eventually, the digestive cell dies in a necrotic manner.

The activation of autophagy and, finally, necrosis has also been observed in *H. dujardini*. The digestive cells of the midgut epithelium are rich in the reserve material, which is primarily represented by proteins, polysaccharides and small amounts of lipids (Hyra et al. 2016). Our studies revealed that the reserve material in *H. dujardini* is enclosed inside the autophagosomes and that it is finally exploited due to autophagy, e.g., lipophagy. Therefore, we can state that selective autophagy is responsible for utilizing the reserve material in this tardigrade species. In the non-infected specimens of *I. g. granulifer*, the cytoplasm of the digestive cells is rich in the reserve material (lipids, proteins, glycoproteins, and glycogen), which can be exploited during oogenesis. It is digested due to autophagy so numerous autophagic structures have been detected in the cytoplasm of the digestive cells during the later stages of oogenesis, which suggests the participation of this accumulated material in vitellogenesis and choriogenesis (Rost-Roszkowska et al. 2011). The reserve material is commonly accumulated in the cytoplasm of the digestive cells in insects (Lipovšek et al. 2011; Amiri and Bandani 2013; Lipovšek and Novak 2015), myriapods (Chajec et al. 2012, 2014; Sosinka et al. 2014), crustaceans (Sousa and Petriella 2000; Sonakowska et al. 2016) and tardigrades (Rost-Roszkowska et al. 2011; Hyra et al. 2016). It can be exploited due to digestion that involves autophagy during the natural periods of starvation such as the hibernation (Lipovšek et al. 2011; Kamińska et al. 2016), molting (Franzetti et al. 2012; Romanelli et al. 2014, 2016) or during starvation that is caused by a lack of food in the environment in which the animal lives (Zaffagnini and Martens 2016). Therefore, it is starvation-induced autophagy, in contrast to a starvation-independent process (Zaffagnini and Martens 2016). During the simplex stage in the tardigrade lifespan, the entire buccal–pharyngeal apparatus is discharged from the body and the animal is able to feed itself. Therefore, the reserve material that has accumulated in the cytoplasm of the digestive cells can be exploited by an animal (Greven 1976; Pirch and Greven 1994; Nelson et al. 2015; Hyra et al. 2016). In all of the specimens of *H. dujardini*, the midgut lumen was devoid of nutrients and the animals that were obtained commercially were not fed. Therefore, we can state that in the case of a lack of food, autophagy participates in the utilization of the reserve material thus supplying the energy for the physiological processes. It is starvation-induced autophagy. If this period is too long and too many autophagic structures accumulate in the cytoplasm, necrosis is involved in the utilization of the entire cell.

We demonstrated that autophagy in the midgut epithelium of Tardigrada, which is the barrier against stressors in these organisms, is a selective process that is responsible for cell survival. Our results showed a distinct crosstalk between autophagy and necrosis in their digestive system, despite the fact that apoptosis has been presented in the other organs of these invertebrates. We also determined that necrosis is the major process that is responsible for the degeneration of the midgut epithelium of tardigrades and, therefore, the “apoptosis–necrosis continuum” (Zeiss 2003), which is the relationship between these two processes, is disrupted.
